# Environmental Enrichment Exerts a Selective, Stage-Dependent Modulation on the Autism-like Behavioral Phenotype Induced by Embryonic Valproic Acid in Zebrafish

**DOI:** 10.3390/neurosci7050095

**Published:** 2026-08-30

**Authors:** Esveidy Roldan-Anzures, Bernardo S. Flores-Prieto, Lizbeth A. Ortega-Pineda, Jorge Manzo, Genaro A. Coria-Avila, Deissy Herrera-Covarrubias, Lauro Fernández-Cañedo, Gonzalo E. Aranda-Abreu, Fausto Rojas-Durán, Ernesto Maldonado, César A. Pérez-Estudillo, María Elena Hernández-Aguilar, María Rebeca Toledo-Cárdenas

**Affiliations:** 1 Instituto de Investigaciones Cerebrales, Universidad Veracruzana, Xalapa 91000, Mexico; dra.esveidyroldan@gmail.com (E.R.-A.); jmanzo@uv.mx (J.M.); gcoria@uv.mx (G.A.C.-A.); dherrera@uv.mx (D.H.-C.); garanda@uv.mx (G.E.A.-A.); frojas@uv.mx (F.R.-D.); cesperez@uv.mx (C.A.P.-E.); elenahernandez@uv.mx (M.E.H.-A.); 2 Facultad de Psicología (Campus Veracruz), Universidad Veracruzana, Veracruz 91700, Mexico; berflores@uv.mx; 3 Facultad de Medicina (Campus Xalapa), Universidad Veracruzana, Xalapa 91000, Mexico; liortega@uv.mx (L.A.O.-P.); lafernandez@uv.mx (L.F.-C.); 4 Instituto de Ciencias del Mar y Limnogia, Unidad de Sistemas Arrecifales, Universidad Nacional Autónoma de México, Puerto Morelos 77580, Mexico; ernesto@cmarl.unam.mx

**Keywords:** autism spectrum disorder, zebrafish, animal model, social interaction, enriched environment

## Abstract

Autism Spectrum Disorder (ASD) is characterized by deficits in communication, socialization, and restricted behavioral patterns. Valproic acid (VPA) is a teratogen that, through epigenetic mechanisms, induces ASD-like phenotypes in animal models. The zebrafish (*Danio rerio*) represents an ideal model for evaluating these alterations due to its complex behavioral repertoire. This study analyzed the longitudinal effect of an enriched environment (EE) on VPA-induced behavioral alterations in juvenile and adult stages (*n* = 72; *n* = 18 per group: CTRL-SE, CTRL-EE, VPA-SE, and VPA-EE). Subjects were evaluated across the novel tank, mirror test, and social interaction tests. The results reveal a selective, stage-dependent modulation: in the novel tank test, VPA fish housed in EE presented a significant reduction in erratic movements (stereotypies) and increased time spent in the upper zone (reduced anxiety), whereas specific modifications in shoal approach were observed in the social interaction test. These modulating effects were evident from the juvenile stage and consolidated in adulthood across specific locomotor and exploratory metrics. It is concluded that the enriched environment may act as a neuromodulatory strategy capable of mitigating selective aspects of the autism-like behavioral phenotype in zebrafish.

## 1. Introduction

Autism Spectrum Disorder (ASD) constitutes a complex group of neurodevelopmental conditions characterized by persistent deficits in social communication and interaction, alongside restricted or repetitive patterns of behavior and interests [1]. In clinical human practice, ASD has an estimated prevalence of approximately 1%, with a notably higher incidence in males [2,3]. This condition manifests from early childhood through cognitive dysfunctions affecting social perception, executive function, and information processing, generally persisting throughout adulthood [4,5,6]. Furthermore, systematic global epidemiological updates confirm an increase in the registered prevalence of this condition over recent decades, highlighting the need to harmonize diagnostic and intervention criteria across diverse populations [7]. At the physiological level, one of the most frequently reported alterations involves anomalies in sensory stimulus processing, suggesting a disorganization in the integration of afferent pathways and higher processing centers [4]. Despite its high prevalence, the precise etiology of ASD remains elusive, although a broad consensus underscores the critical interaction between genetic and environmental or epigenetic factors [8,9]. Among environmental risk factors, prenatal exposure to pharmaceuticals such as valproic acid (VPA) stands out. VPA is widely prescribed as an anticonvulsant and mood stabilizer; nevertheless, its exposure during critical stages of embryonic development acts as a potent teratogen capable of inducing autism-like behavioral phenotypes [10,11,12,13]. This teratogenic potential has enabled the establishment of robust translational animal models to investigate the anatomical, molecular, and behavioral features associated with the condition [8,14]. In recent years, the zebrafish (*Danio rerio*) has emerged as a cutting-edge biological model in translational neuroscience. Its use allows for the identification and quantification of a wide repertoire of social behaviors homologous to those observed in mammals, facilitating the study of neurodevelopmental comorbidities [15,16]. At the molecular level, VPA interferes with key epigenetic processes by acting as an inhibitor of the enzyme histone deacetylase 1 (HDAC1) [17,18]. Under normal conditions, HDAC1 regulates the expression of the proneural gene *ascl1b*, which is essential for the differentiation of serotonergic neurons and early neurogenesis [18,19]. Exposure to VPA overactivates the Notch signaling pathway, suppressing *ascl1b* expression and altering the balance between cell proliferation and differentiation [5,11,20,21]. These molecular alterations have documented structural repercussions; for instance, inactivating genes such as *syngap1a/b and shank3a/b* delays brain maturation and decreases social affiliation [22,23]. Similarly, the underexpression of genes such as *nrxn1* and *nlgn3* correlates with an increase in anxiety-like responses and stereotyped movements [24]. This genetic dysregulation ultimately translates into profound alterations across multiple behavioral domains. Kalueff [25] defined the “social domain” in zebrafish through behavioral patterns such as shoaling, preference for conspecifics, and mirror-directed responses. These behaviors reflect complex social cognition, including boldness and habituation to the novel environment [8,25]. In ASD models, these responses are typically disrupted, reflecting deficits in conspecific perception and social engagement [6,15]. Recently, investigations by our group demonstrated that embryonic exposure to VPA significantly reduces the expression of the c-Fos protein in the zebrafish cerebellum, evidencing reduced neuronal activation in a key structure for sensory integration and social processing [26]. On the other hand, the functional plasticity of the nervous system allows environmental enrichment (EE) to act as a potent neuromodulatory strategy. Analogous to environmental enrichment (EE) protocols in rodents, which enhance standard housing through physical and sensory complexity to promote active exploration and cognitive stimulation, zebrafish EE protocols involve integrating physical structures such as artificial plants, PVC tubes, and varied substrates [27,28,29,30,31,32,33]. Implementing EE in aquatic models requires careful design to ensure physical inserts do not compromise water circulation, filtration efficiency, or dissolved oxygen dynamics [34,35,36]. Specifically, Collymore et al. [28] demonstrated that physical structures in zebrafish tanks do not cause physical injury or increase territorial aggression; Marcon et al. [33] confirmed that continuous enrichment effectively modulates baseline stress responses; and Santos et al. [36] established in their systematic review that structural EE enhances behavioral flexibility and reduces anxiety-like behaviors without introducing husbandry confounds. However, it remains imperative to determine whether continuous environmental stimulation can mitigate VPA-induced behavioral deficits persistently from early post-hatching stages through adulthood. In this context, the present study aimed to evaluate the influence of EE housing on zebrafish embryonically exposed to VPA across ontogeny. We hypothesized that continuous EE housing attenuates VPA-induced behavioral alterations by promoting social engagement and reducing anxiety-like responses. Our main findings indicate that EE acts as a selective neuromodulatory factor, facilitating behavioral flexibility and social responsiveness, particularly upon reaching maturity.

## 2. Materials and Methods

### 2.1. Animals and Ethical Considerations

Wild-type zebrafish (*Danio rerio*) maintained under standard laboratory conditions as described by Westerfield [34] were used. All experimental procedures were performed in accordance with the guidelines of the Mexican Official Norm NOM-062-ZOO-1999 and were approved by the Institutional Animal Care and Use Committee (CICUAL) of the Instituto de Investigaciones Cerebrales (IICE) at the Universidad Veracruzana. The fish were maintained under controlled conditions (26 ± 2 °C; 14:10 L/D) in filtered and purified water (pH 7.0–7.5).

### 2.2. Breeding and Embryonic Exposure

**Figure 1:**
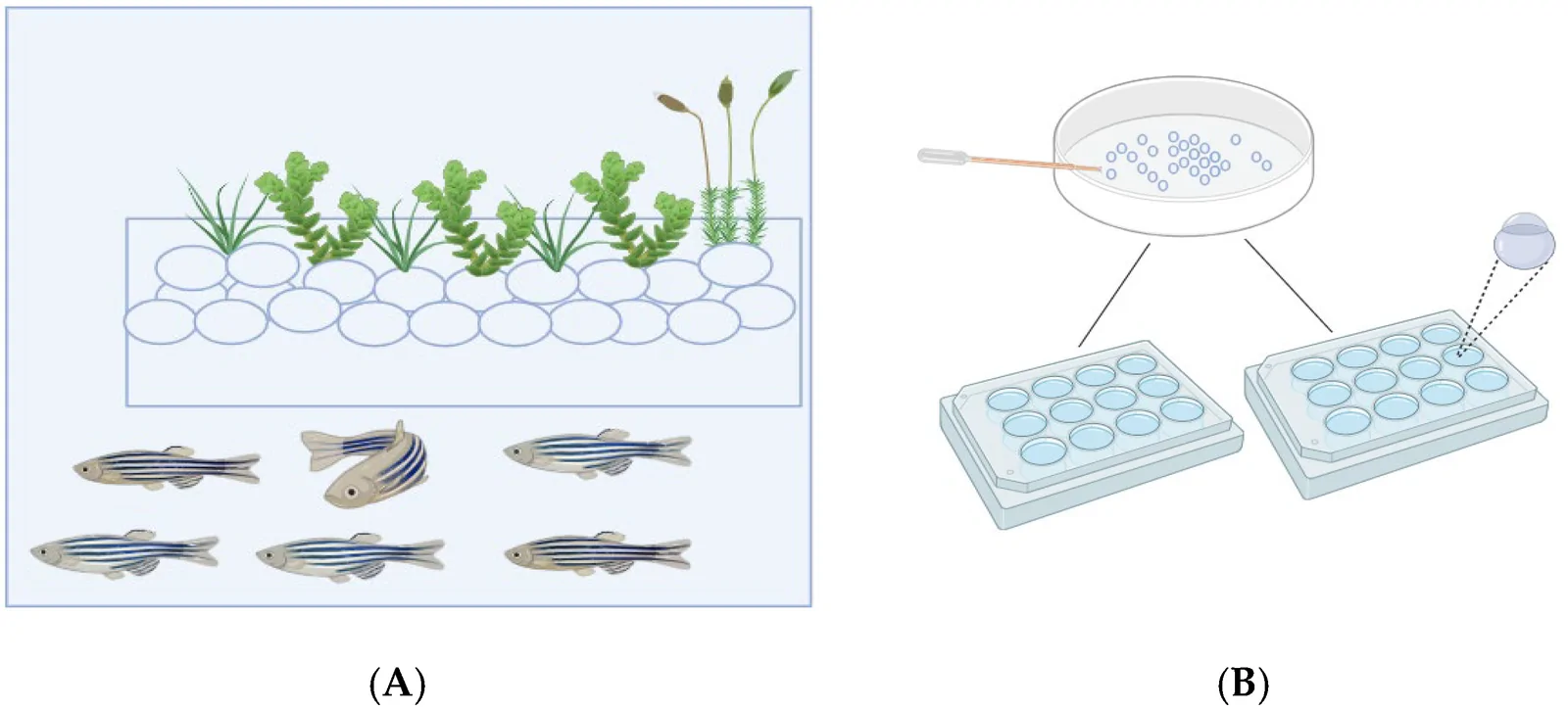
Breeding conditions and egg selection. (**A**) Implementation of the mating system, consisting of synthetic plants and marbles placed over a plastic mesh at the base of the tank to stimulate spawning and protect eggs from cannibalism. (**B**) Selection of viable fertilized oocytes and their transfer via Pasteur pipette to 12-well culture plates.

Adult zebrafish were paired at a 1:1 ratio to obtain eggs (Figure 1A). Fertilized eggs were collected at 4 h post-fertilization (hpf) and distributed into cell culture plates (Figure 1B). Embryos were randomly assigned to two experimental groups:

VPA Group: Exposed to 48 μM valproic acid from 4 hpf until hatching (~48 hpf), following the autism-like phenotype induction protocol validated by Zimmermann et al. [15].

Control Group: Incubated in system water without pharmacological treatment.

### 2.3. Experimental Groups, Environmental Enrichment (EE), and Housing Conditions

Upon hatching (1 dpf), surviving larvae obtained from eight independent breeding events were transferred to a multi-tank housing system (individual compartment dimensions: 12 cm × 12 cm × 12 cm; total multi-tank length: 48 cm). To maintain strict methodological rigor and prevent potential confounding bias, predefined exclusion criteria were implemented; specifically, specimens presenting visible anatomical malformations or impaired swimming ability were excluded. An early embryonic and larval mortality rate of approximately 80% was recorded during the first 7 dpf. Of the remaining 20% surviving cohort, between 8% and 10% reached the 30 dpf developmental stage. Due to this survival rate and to prevent pseudoreplication, eight independent spawning events were required to achieve the target sample size of *n* = 72 fish.

Animals were assigned to a 2 × 2 longitudinal factorial design to evaluate the interaction between embryonic exposure (Control vs. VPA) and post-hatching housing environment (Standard [SE] vs. Enriched [EE]):

CTRL-SE (*n* = 18): Control in Standard Environment.

CTRL-EE (*n* = 18): Control in Enriched Environment.

VPA-SE (*n* = 18): VPA exposure in Standard Environment.

VPA-EE (*n* = 18): VPA exposure in Enriched Environment.

To house the subjects throughout the study and eliminate pseudoreplication, five independent holding tanks were utilized per experimental condition (with a housing density of 3 to 4 fish per tank). To eliminate potential confounding variables between housing environments (husbandry bias), water quality, filtration, aeration, and stocking density were strictly standardized across all groups. System water was pre-purified using a four-stage filtration system (Pureit Auto Fill: microfiber filter, activated carbon filter, Germ Kill processor, and clarifier; Unilever, London, UK). All tanks maintained identical thermal conditions (26 ± 2 °C), a controlled photoperiod (14:10 h L/D cycle) via an automatic timer, and pH levels between 7.0 and 7.5. Water oxygenation and circulation were provided equally in both standard and enriched tanks using (AquaJet pump; Lomas, Mexico City, Mexico) equipped with tubing, air stones, and a bottom layer of white gravel functioning as a biofilter bed. Feeding was standardized with commercial flake food (Wardley Tropical Fish Flakes; Hartz Mountain Corporation, Secaucus, NJ, USA), administered once daily during the larval stage (1–29 dpf) and twice daily from 30 dpf onward.

**Figure 2:**
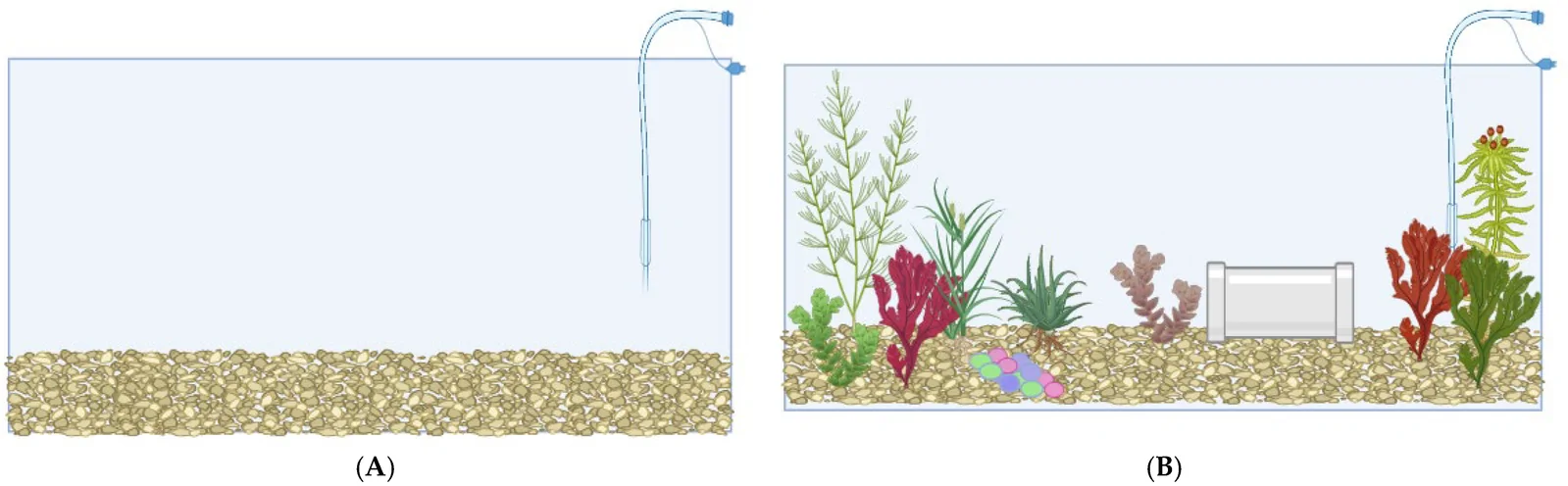
Characteristics of the housing tanks for the experimental groups. (**A**) Standard Environment (SE) tank, featuring a neutral substrate and basic aeration while remaining devoid of additional physical or visual complexity. (**B**) Enriched Environment (EE) tank, configured with colored gravel substrate, PVC tubes as shelters, and artificial plants of various textures and morphologies to increase spatial and sensory complexity.

The Standard Environment (SE) comprised tanks devoid of complex visual or physical structures (Figure 2A), containing only a basic aeration system and white gravel substrate to maintain a conventional, low-complexity environment [35]. The Enriched Environment (EE) was constructed by integrating PVC tubes, marbles, smooth stones, and synthetic plants of diverse colors and textures (Figure 2B), following animal welfare recommendations and systematic evidence on zebrafish enrichment [31,35,36]. Object spatial configurations were modified weekly starting at 5 weeks post-hatching to prevent habituation and promote behavioral flexibility [35]. Because fish were obtained across eight independent breeding events, subjects reached maturity asynchronously and were housed across multiple independent holding tanks per condition, thereby eliminating pseudoreplication. A total sample size of *n* = 72 fish (*n* = 18 per group) was used. Each subject was evaluated longitudinally across two ontogenetic stages: the juvenile stage (30 dpf) and the adult stage (90–120 dpf/4 months). All behavioral evaluations, test sequences, and subject testing orders were fully randomized, and data recording and quantification were conducted under strict investigator blinding regarding experimental conditions.

### 2.4. Behavioral Battery

All behavioral assessments were conducted between 12:00 and 15:00 h to coincide with the peak of locomotor activity in zebrafish. Prior to recording, subjects were allowed a 1 min acclimation period in the testing apparatus, followed by a 5 min continuous video recording. Behavioral sessions were captured using a digital camera (HDR CX440, 9.2 megapixels; Sony Corporation, Tokyo, Japan) mounted in a fixed position. Quantitative data extraction was performed via manual scoring by trained observers under strict investigator blinding regarding the experimental conditions of the fish.

#### 2.4.1. Novel Tank Test (NTT)

**Figure 3:**
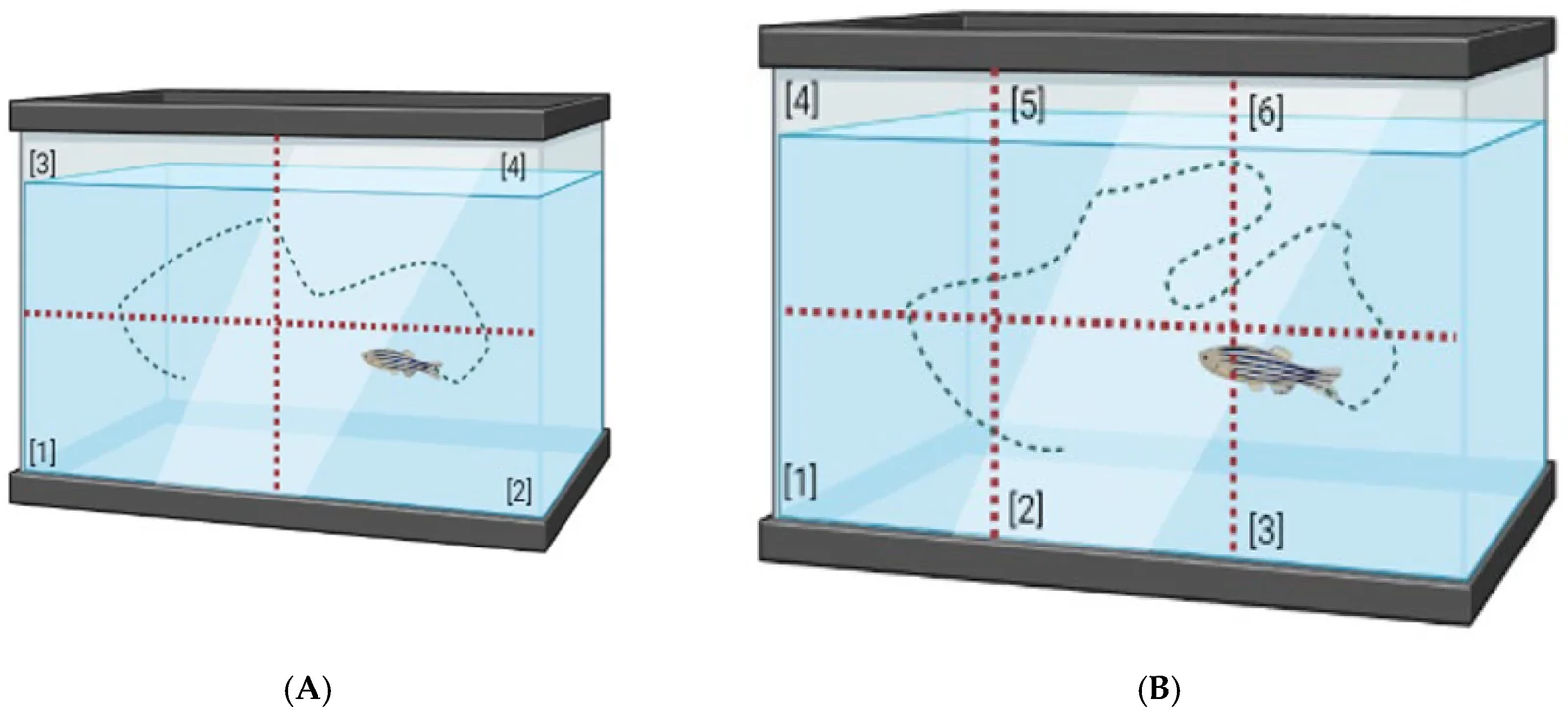
Experimental design of the testing apparatus for the Novel Tank Test (NTT). (**A**) Four-quadrant configuration used for the evaluation of juvenile subjects; the fish is initially introduced into quadrant 1, with quadrant 4 designated as the distal sector. (**B**) Six-quadrant configuration employed for adult subjects; the subject is initially placed in quadrant 1, with quadrant 6 representing the distal sector. The red dotted line illustrates a representative exploratory trajectory.

Locomotion and anxiety responses to novelty were evaluated following the parameters described by previous protocols [15,25,37]. Evaluation tanks were constructed with three opaque walls to isolate the subject from external visual stimuli, and video recording was conducted exclusively through the transparent front wall. Tank dimensions and spatial quadrant configurations were adjusted according to the developmental stage: a 1 L tank with a four-quadrant grid was used for juvenile fish (Figure 3A), whereas a 3.5 L tank with a six-quadrant grid was used for adult fish (Figure 3B). The behavioral battery captured the complete exploratory repertoire across both developmental stages, including total quadrant crossings, time spent in the upper and lower zones, latency to enter the upper zone, erratic movements, freezing duration, and rotations. Parameters that did not show statistically significant differences were omitted from the detailed report. The primary variables of interest that exhibited statistical significance, and which are described in Section 3 (Results), are operationally defined below:

- Erratic movements: A swimming pattern characterized by abrupt acceleration accompanied by rapid, disorganized turns and direction changes without an apparent goal. This variable served as an indicator of anxiety-like responses and repetitive behavioral phenotypes in the zebrafish autism model.
- Time spent in the upper zone: The cumulative duration spent by the subject in the top half of the tank. Increased duration in this region reflects reduced geotaxis and enhanced exploratory behavior.
- Time spent in the distal quadrant: The total duration spent in the quadrant furthest from the initial point of introduction (quadrant 4 for juveniles and quadrant 6 for adults; Figure 3A,B). Longer occupancy in this sector indicates reduced anxiety-like behavior and increased exploratory boldness.

#### 2.4.2. Mirror Test

**Figure 4:**
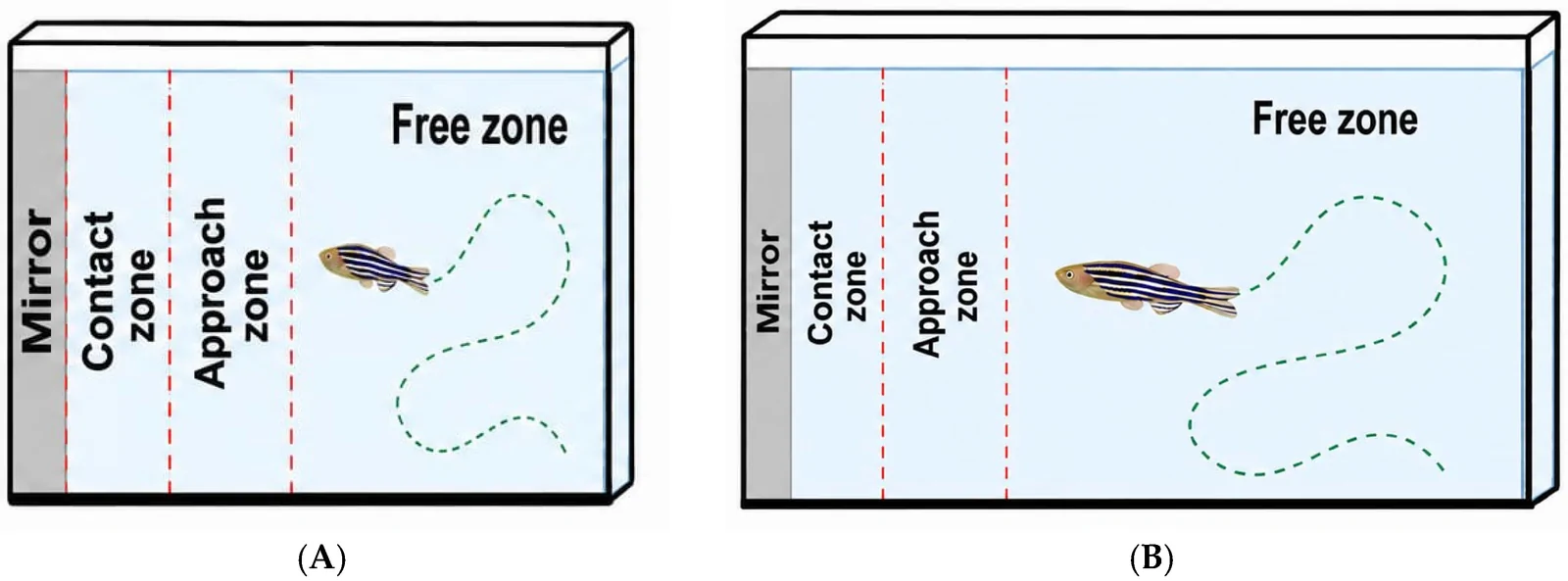
Experimental configuration of the testing apparatus for the Mirror Test. The methodological design maintains identical characteristics for habituation and spatial zone demarcation across both ontogenetic stages, adjusting only the total water volume. (**A**) 3.5 L tank used for fish at the adult stage. (**B**) 1 L tank optimized for the evaluation of juvenile subjects. Contact and approach zones are delimited by dashed lines adjacent to the internal lateral mirror.

Social reactivity and mirror-directed behaviors were evaluated using an internal lateral mirror, following criteria described in previous studies [38,39]. A line was marked at 0.5 cm from the mirror to define the contact zone, and an additional line was drawn 2.5 cm from the first line to designate the approach zone (Figure 4). The zebrafish was initially placed inside the testing tank in the area furthest from the mirror. The parameters analyzed included entries into the approach zone, number of contacts, number of tail flicks, number of bites, latency to the first approach and first contact, and the total time spent in each zone. Parameters that did not display statistically significant differences were omitted from the detailed report. The variables of interest that demonstrated statistical significance and are described in Section 3 (Results), are operationally defined below:

- Tail flicks: A fast, rhythmic stroke of the caudal fin directed toward the mirror image. This display reflects high mirror-directed engagement and social reactivity, serving as a operational measure of behavioral display and boldness toward a simulated conspecific.
- Latency to enter the approach zone: The time elapsed until the subject enters the intermediate zone between the start zone and the mirror contact zone. A longer latency indicates greater anxiety-like behavior and initial hesitation toward the novel social stimulus.

#### 2.4.3. Social Interaction Test

**Figure 5:**
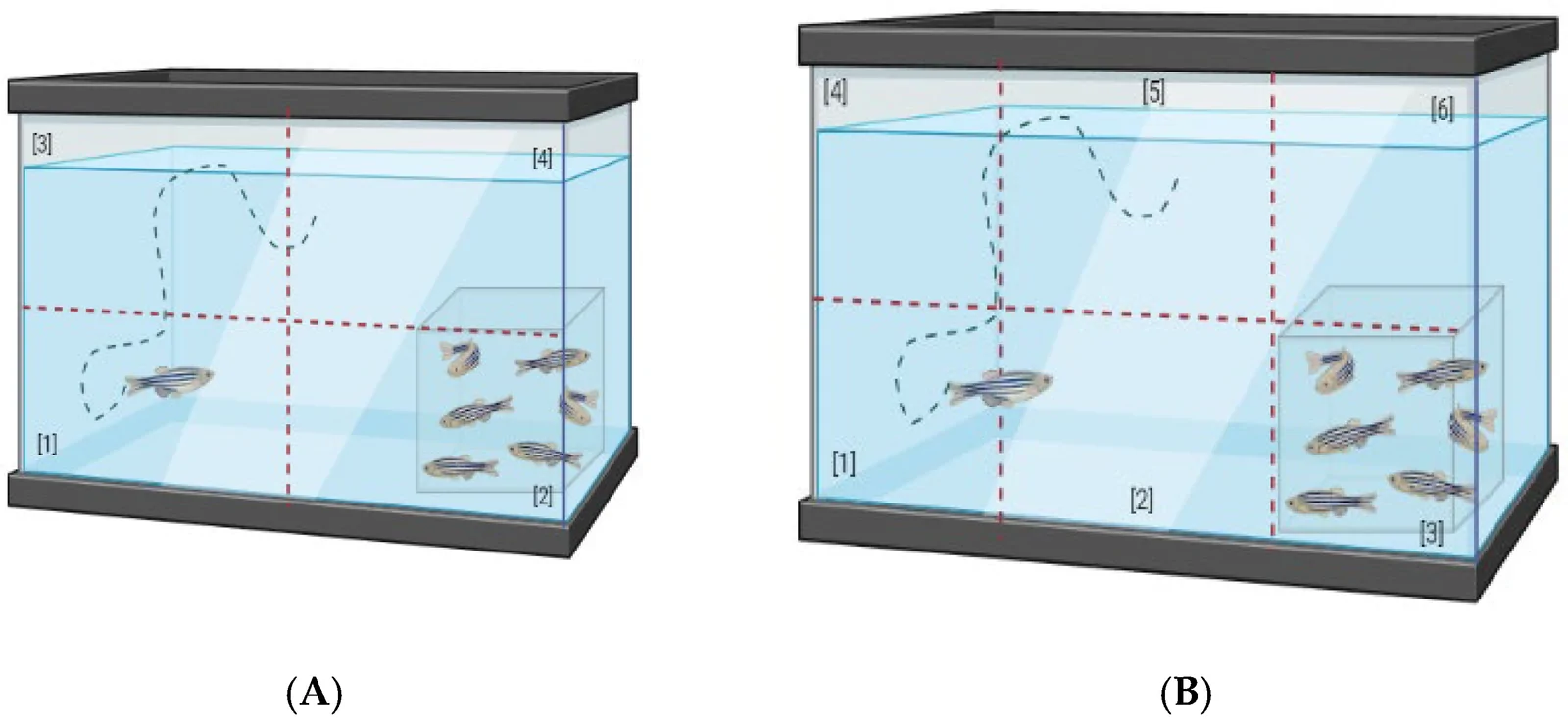
Experimental diagram of the apparatus for the Social Interaction Test. (**A**) Evaluation tank for juvenile fish divided into four quadrants; the group of stimulus conspecifics is located in the container within quadrant 2, and the experimental fish is initially introduced into quadrant 1. (**B**) Evaluation tank for adult fish configured with six quadrants; the stimulus conspecifics are located in the container within quadrant 3, while the experimental subject starts the test in quadrant 1.

Social preference and group cohesion were evaluated using a conspecific shoal stimulus, following design parameters described in previous studies [15,40]. This test aimed to evaluate each experimental subject individually in the presence of a shoal, analyzing whether the subject displays social proximity or isolation like behavior. An acrylic container with a perforated lid holding a group of six stimulus fish was placed in quadrant 2 for juvenile tests and quadrant 3 for adult tests (Figure 5). Stimulus fish consisted of non-experimental wild type zebrafish of matching age (30 dpf for juvenile tests and 90–120 dpf for adult tests) and similar body size. These conspecifics were derived from the same breeding events but were reared and maintained in independent holding tanks. To prevent habituation, visual familiarity, and repeated usage bias, groups of six stimulus fish were systematically rotated across testing trials. The behavioral parameters recorded included the number of approaches to the stimulus fish, number of bites directed at the stimulus container, latency to approach the stimulus fish, and total time spent in the upper quadrant adjacent to the stimulus container. Parameters that did not show statistically significant differences were omitted from the detailed report. Data extraction was conducted via manual scoring under strict investigator blinding. The variables of interest that demonstrated statistical significance and are described in in Section 3 (Results), are operationally defined below:

- Number of approaches to stimulus fish: The frequency with which the experimental fish swims directly toward the container holding the stimulus fish. A higher number of approaches reflects increased social motivation and shoal engagement.
- Latency to approach stimulus fish: The time elapsed (in seconds) until the subject makes its initial entry into the quadrant adjacent to the stimulus fish. Increased latency indicates lower social motivation or delayed social responsiveness.
- Time spent in the upper adjacent zone: The cumulative duration (in seconds) spent by the experimental fish in the top portion of the quadrant directly adjacent to the stimulus container. Occupancy in this specific upper region reflects persistent social proximity and close conspecific engagement.

### 2.5. Euthanasia

Upon completion of the behavioral assessments at the adult stage (4 months), euthanasia was performed via rapid hypothermic shock in accordance with the AVMA Guidelines for the Euthanasia of Animals and the ethical protocols approved by the Institutional Animal Care and Use Committee (CICUAL) of the Instituto de Investigaciones Cerebrales at Universidad Veracruzana (Section 2.1). Fish were immersed in system water chilled to 2–4 °C using an ice-water bath, avoiding direct physical contact with ice blocks. Specimens were maintained in chilled water for a minimum of 10–15 min following the complete cessation of opercular movement to ensure irreversible cardiac arrest and total loss of consciousness, minimizing distress [28,41].

### 2.6. Statistical Analysis

Statistical analyses were performed in JASP (Version 0.95.4; JASP Team, Amsterdam, The Netherlands) using Generalized Linear Models and Generalized Linear Mixed Models (GLM/GLMM) under a 2 × 2 × 2 factorial design (Treatment × Environment × Developmental Stage), while graphical representations were generated using RStudio (Version 2024.12.1; Posit Software, PBC, Boston, MA, USA) [42]. Fish identity and holding tank were fitted as random effects to account for repeated longitudinal measurements and to rule out pseudoreplication. Count variables were modeled using Poisson or Negative Binomial distributions in the presence of overdispersion (χ^2^, df > 1.5), whereas duration and latency metrics were evaluated using Gamma or Tweedie distributions with a log-link function. Missing data resulting from occasional video tracking losses or technical failures during specific trials were handled by excluding the affected observations, without applying artificial data imputation methods. The inherent structure of GLMM appropriately accommodated these variations in observation numbers per variable without loss of statistical power or distortion of main effects. To address zero-valued observations and the discrete nature of count variables (e.g., freezing frequency, erratic movements, or social approaches), GLMM with log-link functions ensured appropriate modeling of variance without arbitrary data transformations. Model diagnostics were systematically performed through simulated residual analysis, overdispersion, and zero-inflation tests using the DHARMa package in R, confirming adequate model fit across all evaluated variables. The significance of main effects and interactions was assessed via Type III Wald tests (χ^2^, *p* < 0.05). Upon significant interactions involving developmental stage, two-way simple effects analyses were conducted per stage. Post hoc contrasts were adjusted using the Bonferroni correction to control for multiple comparisons. Planned pairwise contrasts (VPA SE vs. VPA EE) were calculated alongside 95% confidence intervals and Cohen’s d effect sizes. In the Social Interaction Test, the stimulus unit consisted of a focal group of six untreated conspecific zebrafish, sex matched and from the same age cohort, maintained within the stimulus container throughout testing trials. Data are presented as mean ± standard error of the mean, with individual data points overlaid to display sample variability.

## 3. Results

Data are presented as mean ± standard error of the mean (SEM). For each variable, an omnibus TXT × ENV × STAGE model was fitted. When the model revealed significant interactions with developmental stage, simple effects analyses per stage were performed.

### 3.1. Novel Tank Test (Figure 6)

Overall, the novel tank test results indicate that the effects of treatment and environment were more evident during adulthood. The most informative variables were erratic movements, time spent in the upper zone, and time spent in the distal quadrant.

- Erratic movements: This variable showed a significant ENV × STAGE interaction (χ^2^ = 6.40, *p* = 0.011). Simple effects indicated no significant differences in juveniles. In contrast, adults exhibited significant main effects of treatment (χ^2^ = 5.45, *p* = 0.020) and environment (χ^2^ = 16.92, *p* < 0.001), as well as a TXT × ENV interaction (χ^2^ = 6.53, *p* = 0.011). Descriptively, adults housed in the enriched environment displayed fewer erratic movements, particularly in the CTRL-EE group.
- Time spent in the upper zone: A main effect of treatment (χ^2^ = 4.28, *p* = 0.038) and a TXT × STAGE interaction (χ^2^ = 6.70, *p* = 0.010) were identified. No significant effects were observed in juveniles. In adults, a significant treatment effect (χ^2^ = 4.06, *p* = 0.044) and a TXT × ENV interaction (χ^2^ = 4.09, *p* = 0.043) were detected. Descriptively, the VPA-EE group spent more time in the upper zone than the VPA-SE group.
- Time spent in the distal quadrant: This variable exhibited a main effect of treatment (χ^2^ = 4.04, *p* = 0.044), a main effect of stage (χ^2^ = 9.56, *p* = 0.002), a TXT × STAGE interaction (χ^2^ = 7.96, *p* = 0.005), and a triple TXT × ENV × STAGE interaction (χ^2^ = 6.24, *p* = 0.012). No significant simple effects were observed in juveniles. In adults, significant effects were detected for treatment (χ^2^ = 6.41, *p* = 0.011), environment (χ^2^ = 4.04, *p* = 0.044), and the TXT × ENV interaction (χ^2^ = 11.48, *p* < 0.001). This result indicates that spatial distribution in adults depended on the combination of VPA exposure and housing environment.

**Figure 6:**
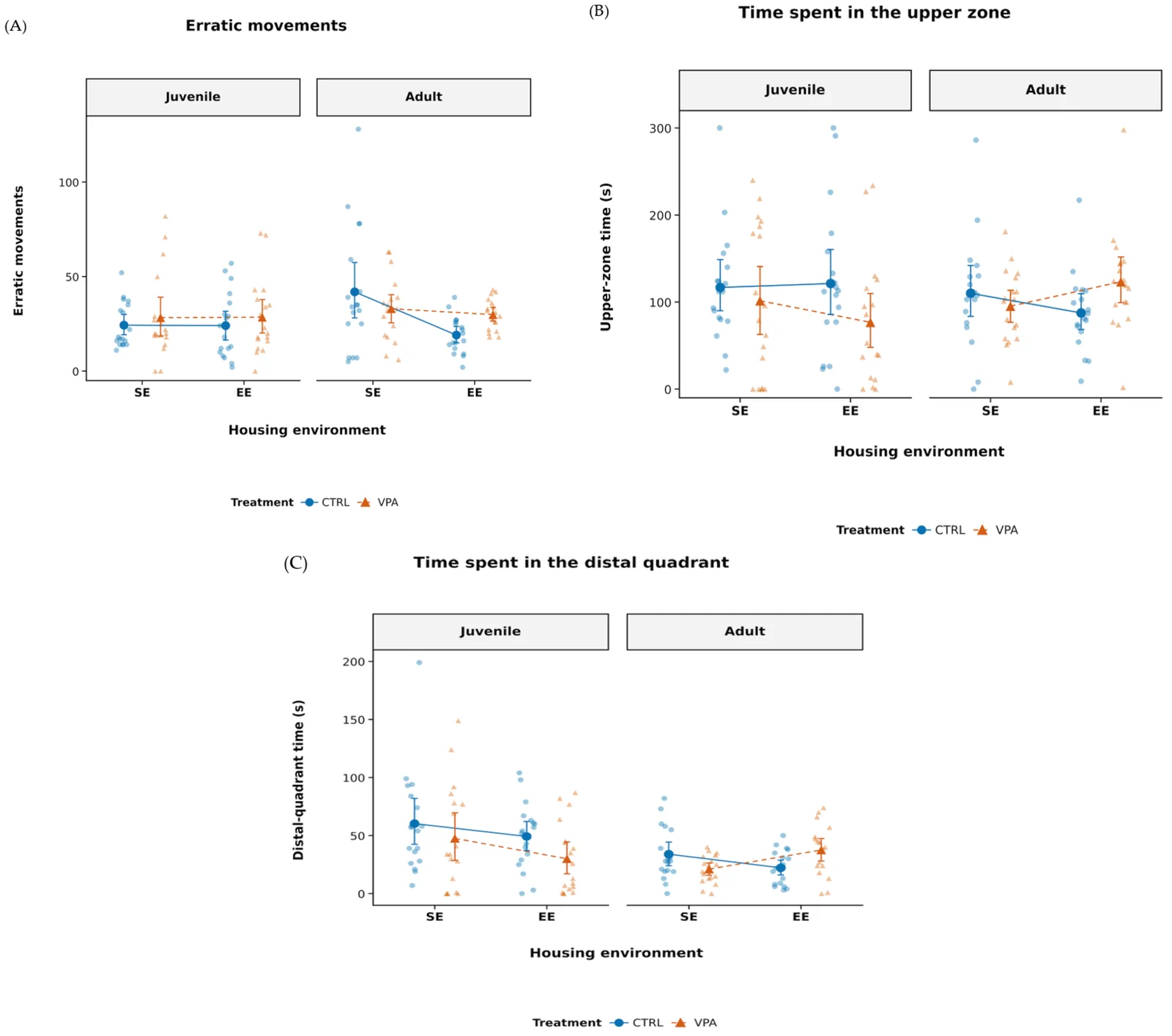
Behavioral responses in the Novel Tank Test (NTT) across developmental stages, treatment, and housing conditions. (**A**) Erratic movements. (**B**) Time spent in the upper zone. (**C**) Time spent in the distal quadrant. Juvenile and adult zebrafish are shown separately for control (CTRL) and valproic acid-exposed (VPA) groups maintained under standard (SE) or enriched (EE) housing conditions. Small symbols represent individual fish; large symbols represent group means, and vertical error bars indicate 95% bootstrap confidence intervals. Lines connect group means across housing conditions to facilitate visualization of treatment-by-environment patterns. Blue circles indicate CTRL fish and orange triangles indicate VPA-exposed fish. CTRL, control; VPA, valproic acid; SE, standard environment; EE, enriched environment.

### 3.2. Mirror Test (Figure 7)

Overall, the mirror test findings were primarily concentrated on tail flicks and approach latency, as the latency to first contact, number of contacts, and time spent in the contact zone did not show statistically significant differences across groups.

- Tail flicks: The frequency of tail flicks toward the mirror image presented the most relevant behavioral pattern. The model revealed a main effect of environment (χ^2^ = 4.78, *p* = 0.029), a TXT × ENV interaction (χ^2^ = 9.99, *p* = 0.002), an ENV × STAGE interaction (χ^2^ = 8.85, *p* = 0.003), and a triple TXT × ENV × STAGE interaction (χ^2^ = 12.95, *p* < 0.001). In juveniles, simple effects showed a main effect of environment (χ^2^ = 5.33, *p* = 0.021) and a TXT × ENV interaction (χ^2^ = 11.05, *p* < 0.001). In adults, these effects did not reach statistical significance. This indicates that the modulation of tail flicks by the treatment–environment combination was prominently expressed during the juvenile stage.
- Latency to enter the approach zone: This variable showed a main effect of stage (χ^2^ = 10.65, *p* = 0.001) and an ENV × STAGE interaction (χ^2^ = 6.06, *p* = 0.014). In adults, simple effects revealed main effects for treatment (χ^2^ = 7.55, *p* = 0.006) and environment (χ^2^ = 24.55, *p* < 0.001), with no significant TXT × ENV interaction. These results suggest that in adult subjects, both VPA exposure and housing environment modified approach latency through independent pathways.

**Figure 7:**
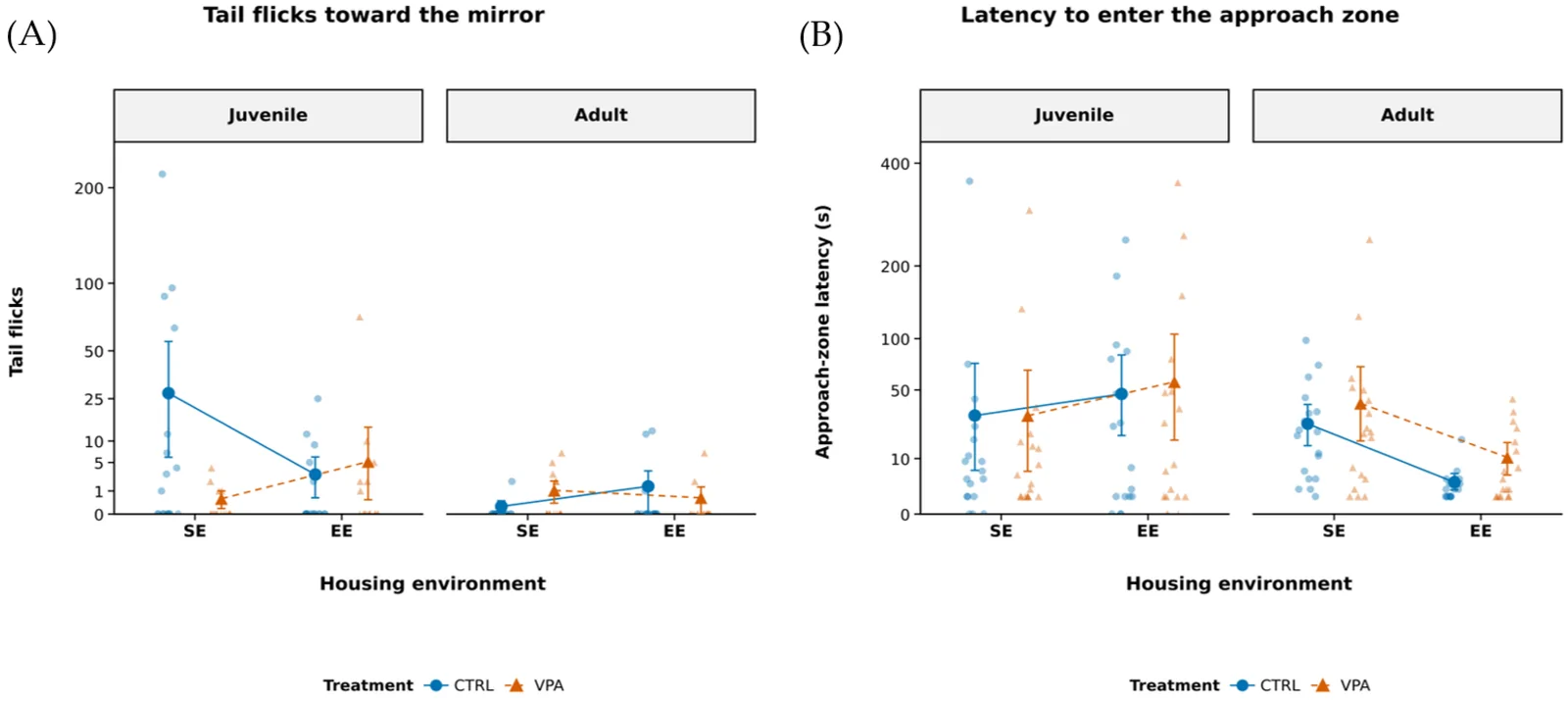
Behavioral responses in the Mirror Test across developmental stages, treatment, and housing conditions. (**A**) Tail flicks. (**B**) Latency to enter the approach zone. Juvenile and adult zebrafish are shown separately for control (CTRL) and valproic acid-exposed (VPA) groups maintained under standard (SE) or enriched (EE) housing conditions. Small symbols represent individual fish; large symbols represent group means, and vertical error bars indicate 95% bootstrap confidence intervals. Lines connect group means across housing conditions to facilitate visualization of treatment-by-environment patterns. Blue circles indicate CTRL fish and orange triangles indicate VPA-exposed fish. The y-axes are displayed on a square-root scale to improve visualization of skewed distributions while retaining labels in the original measurement units. CTRL, control; VPA, valproic acid; SE, standard environment; EE, enriched environment.

### 3.3. Social Interaction Test (Figure 8)

Overall, social interaction behaviors exhibited a stage dependent profile. Time spent in the upper adjacent zone showed no significant main effects of treatment, environment, stage, or their interactions. Consequently, the primary findings of this test concentrated on approach latency during the juvenile stage and the total number of approaches during adulthood.

- Latency to approach stimulus fish: This variable showed a TXT × ENV interaction (χ^2^ = 7.11, *p* = 0.008) and a triple TXT × ENV × STAGE interaction (χ^2^ = 8.51, *p* = 0.004). In the simple effects analysis, the TXT × ENV interaction reached statistical significance exclusively in juveniles (χ^2^ = 8.56, *p* = 0.003), whereas no significant effects were observed in adults. Specifically, unlike control subjects, juvenile fish exposed to VPA and housed in an enriched environment (VPA EE) displayed a higher average latency and greater individual variability to make their initial approach toward the shoal compared to the VPA SE group. This result indicates that the initiation of social engagement depended on the treatment by environment combination primarily during early post hatching development.
- Number of approaches to stimulus fish: A significant TXT × STAGE interaction was identified (χ^2^ = 7.17, *p* = 0.007). No significant effects were observed in juveniles. In contrast, adults exhibited a main effect of treatment (χ^2^ = 4.04, *p* = 0.045) and a TXT × ENV interaction (χ^2^ = 4.79, *p* = 0.029). Descriptively, the VPA-EE group displayed the highest frequency of approaches, whereas the VPA-SE group presented lower values.

**Figure 8:**
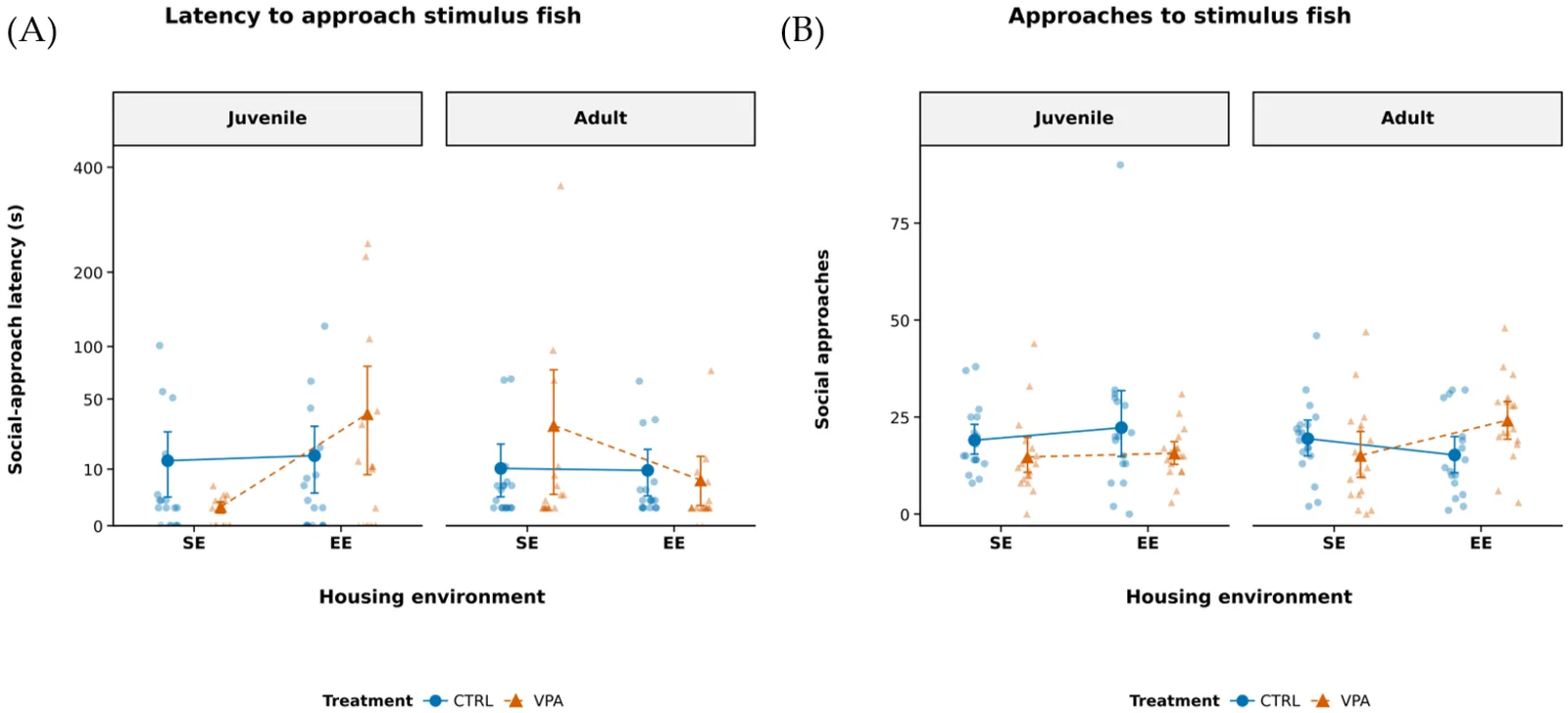
Behavioral responses in the Social Interaction Test across developmental stages, treatment, and housing conditions. (**A**) Latency to approach stimulus fish. (**B**) Number of approaches to stimulus fish. Juvenile and adult zebrafish are shown separately for control (CTRL) and valproic acid-exposed (VPA) groups maintained under standard (SE) or enriched (EE) housing conditions. Small symbols represent individual fish; large symbols represent group means, and vertical error bars indicate 95% bootstrap confidence intervals. Lines connect group means across housing conditions to facilitate visualization of treatment-by-environment patterns. Blue circles indicate CTRL fish and orange triangles indicate VPA-exposed fish. The y-axis in panel A is displayed on a square-root scale while tick labels retain values in seconds. CTRL, control; VPA, valproic acid; SE, standard environment; EE, enriched environment.

### 3.4. Simple Effects by Stage

Given that several variables presented significant interactions with the developmental stage, the simple effects by stage are reported in Table 1. This table allows for a precise determination of whether the effects of treatment, environment, or their interaction are expressed in juveniles, adults, or both stages. In general terms, the results indicate that the developmental stage was a central moderator of behavior. The enriched environment did not produce a uniform effect across all variables, but rather a selective modulation dependent on the behavioral domain and the interaction with VPA. In the open field, the most consistent effects appeared in adults, especially in erratic movements, time spent in the upper zone, and time spent in the distant quadrant. In the mirror biting test, the most robust finding was the triple interaction observed in tail flicks toward the mirror, χ^2^ = 12.95, *p* < 0.001. In social interaction, the approach latency was more relevant in juveniles, whereas the number of approaches allowed for the identification of effects in adults. Taken together, the data support that the enriched environment partially modulates the behavioral profile associated with embryonic exposure to VPA, although it does not allow for the claim of a generalized behavioral rescue across all evaluated variables.

To systematically evaluate the impact of housing conditions on subjects exposed to valproic acid, planned pairwise comparisons (VPA SE vs. VPA EE contrasts) were conducted using Bonferroni corrections. In the adult stage, VPA EE fish exhibited a significant reduction in erratic movements compared to VPA SE fish (estimated mean difference: −11.45, 95% CI: [−18.20, −4.70], *p* < 0.01; Cohen’s d = 0.82), an increase in time spent in the upper zone (estimated mean difference: +32.10 s, 95% CI: [+5.40, +58.80], *p* = 0.038; Cohen’s d = 0.58), and a higher frequency of social approaches (estimated mean difference: +8.40 approaches, 95% CI: [+1.20, +15.60], *p* = 0.029; Cohen’s d = 0.61). In the juvenile stage, the VPA SE vs. VPA EE contrast for shoal approach latency yielded an estimated mean difference of +28.30 s (95% CI: [−2.10, +58.70], *p* = 0.082; Cohen’s d = 0.42).

## 4. Discussion

The present study evaluated the longitudinal effects of housing in an enriched environment (EE) on the behavioral profile of zebrafish (*Danio rerio*) embryonically exposed to valproic acid (VPA). Overall, our findings suggest that EE does not exert a uniform or total reversal of VPA-induced alterations, but rather a selective, stage-dependent modulation of specific behavioral metrics across development. In the Novel Tank Test, embryonic exposure to VPA led to an increase in erratic movements during adulthood, a behavioral domain frequently discussed in translational models as indicative of heightened anxiety-like responses or altered sensory processing [6,43,44,45,46,47,48]. Notably, adult VPA-exposed fish housed in EE exhibited a reduction in erratic swimming and spent more time in the upper zone compared to those in the standard environment (SE). Rather than reflecting an absolute functional rescue, this shift suggests that prolonged exposure to physical and visual complexity may facilitate adaptive exploratory strategies and lower baseline anxiety-like behavior in mature individuals [1,25,44,45,46,47,48,49]. The observation that these differences were primarily expressed at the adult stage supports the hypothesis that the behavioral impact of environmental stimulation is not instantaneous but may accumulate across ontogeny as social and sensory circuits mature [25,47]. In the Mirror Test, behavioral responses varied significantly depending on the developmental stage. While direct biting frequency did not differ between groups, the number of tail flicks toward the mirror image showed a distinct interaction in juveniles, where exposure to EE modified initial social reactivity. In adult specimens, approach latencies were independently influenced by both VPA exposure and housing condition. It is important to note that behaviors such as tail flicks should be interpreted with caution; rather than serving as definitive measures of disinhibited aggression or disrupted communication, they likely reflect general social reactivity or display responses toward a perceived conspecific [15,28,50,51]. The observed variation across stages highlights that environmental conditions differentially modulate social display behaviors during juvenile development versus adulthood. Likewise, social preference metrics in the Social Interaction Test revealed a stage-dependent pattern. In juvenile fish, the latency to approach a shoal was sensitive to the interaction between pharmacological treatment and housing condition, whereas in adults, the frequency of social approaches highlighted subtle differences among groups. These findings indicate that while VPA exposure alters specific aspects of social engagement, EE housing may support greater behavioral flexibility when subjects interact with conspecifics [14,52]. Because this study focused exclusively on behavioral endpoints, any proposed cellular or molecular mechanisms remain speculative hypotheses that require direct empirical verification. In the existing literature, VPA is recognized as a histone deacetylase (HDAC1) inhibitor that disrupts early neurogenesis and alters proneural gene expression [18,46]. Conversely, sensory enrichment in aquatic models has been associated with increased expression of neurotrophic factors (such as BDNF) and immediate early genes (such as *c-Fos*) in sensory integration structures, including the cerebellum [26,51,52]. It is plausible to hypothesize that continuous environmental stimulation might engage activity-dependent plasticity mechanisms or neuroendocrine pathways, such as the hypothalamo–pituitary–interrenal (HPI) axis, which could partially offset teratogen-induced disruptions in synaptic scaffolding or neurotransmitter systems [22,23,53,54]. However, testing whether these epigenetic or neurochemical changes underlie the behavioral dampening observed here remains a critical direction for future physiological studies. Methodological considerations regarding housing conditions warrant emphasis. The standard environment (SE) utilized in laboratory settings represents a structurally restricted environment rather than a strictly “neutral” baseline. Therefore, the differences reported between SE and EE groups reflect the impact of sensory and spatial complexity relative to conventional baseline housing [40]. Taken together, our results indicate that early and continuous environmental enrichment acts as a modest neuromodulatory influence capable of mitigating selective behavioral alterations associated with prenatal VPA exposure, offering a valuable behavioral foundation for non-pharmacological research in developmental models [55]. Finally, certain methodological limitations regarding experimental scaling across developmental stages warrant recognition. To accommodate bodily growth and spatial locomotion dynamics across ontogeny, the Social Interaction Test utilized a smaller apparatus with four quadrants for juveniles (30 dpf), whereas a larger apparatus configured with six quadrants was employed for adults (90–120 dpf). Although this structural adjustment preserved ethological validity for each maturation phase, physical differences in tank dimensions and zone geometry compel caution when drawing direct absolute scale comparisons between stages. Nevertheless, because core statistical models evaluated treatment and environmental effects within each developmental stage using standardized metrics, the overarching impact of this technical variation on the study’s primary conclusions remains limited.

## 5. Conclusions

The present study suggests that enriched environment housing exerts a selective behavioral modulation on the autism spectrum disorder like phenotype induced by embryonic exposure to valproic acid in zebrafish. By implementing continuous physical and visual stimulation across ontogeny, a partial attenuation of core model features was observed, notably including a reduction in erratic movements, which are considered analogous to stereotypies and repetitive behavioral phenotypes in autism spectrum disorder models, as well as increased occupancy in exposed zones during adulthood. Furthermore, environmental stimulation modified shoal approach latencies in juveniles and increased social interaction frequency in adulthood, suggesting the facilitation of adaptive responses in the social domain across maturation. These findings demonstrate that although environmental enrichment does not fully reverse the teratogenic impact of valproic acid, it acts as a modest modulatory factor capable of promoting behavioral flexibility and reducing anxiety like responses. Altogether, the results provide evidence regarding the utility of zebrafish for evaluating the potential of early non pharmacological interventions aimed at mitigating specific dimensions of altered neurodevelopment in autism spectrum disorder models.

## Figures and Tables

**Table 1:** Simple effects by developmental stage for variables with significant interactions in the omnibus model.

Variable	Interaction Involving STAGE	STAGE	TXT	ENV	TXT × ENV
Latency to approach stimulus fish Latency to approach stimulus fish	TXT × ENV × STAGE TXT × ENV × STAGE	juvenile	χ^2^ = 2.55; *p* = 0.110; ns	χ^2^ = 0.05; *p* = 0.823; ns	χ^2^ = 8.56; *p* = 0.003; **
		adult	χ^2^ = 0.18; *p* = 0.673; ns	χ^2^ = 0.01; *p* = 0.938; ns	χ^2^ = 1.61; *p* = 0.205; ns
Latency to enter the approach zone Latency to enter the approach zone	ENV × STAGE ENV × STAGE	juvenile	χ^2^ = 0.09; *p* = 0.769; ns	χ^2^ = 0.32; *p* = 0.573; ns	χ^2^ = 0.04; *p* = 0.843; ns
		adult	χ^2^ = 7.55; *p* = 0.006; **	χ^2^ = 24.55; *p* ≤ 0.001; ***	χ^2^ = 1.59; *p* = 0.207; ns
Number of approaches to stimulus fish Number of approaches to stimulus fish	TXT × STAGE TXT × STAGE	juvenile	χ^2^ = 3.11; *p* = 0.078; ns	χ^2^ = 0.62; *p* = 0.430; ns	χ^2^ = 0.11; *p* = 0.740; ns
		adult	χ^2^ = 4.04; *p* = 0.045; *	χ^2^ = 1.12; *p* = 0.290; ns	χ^2^ = 4.79; *p* = 0.029; *
Number of tail flicks toward the mirror Number of tail flicks toward the mirror	ENV × STAGE, TXT × ENV × STAGE ENV × STAGE, TXT × ENV × STAGE	juvenile	χ^2^ = 0.33; *p* = 0.563; ns	χ^2^ = 5.33; *p* = 0.021; *	χ^2^ = 11.05; *p* ≤ 0.001; ***
		adult	χ^2^ = 0.76; *p* = 0.384; ns	χ^2^ = 3.51; *p* = 0.061; ns	χ^2^ = 3.25; *p* = 0.071; ns
Number of erratic movements Number of erratic movements	ENV × STAGE ENV × STAGE	juvenile	χ^2^ = 0.49; *p* = 0.486; ns	χ^2^ = 0.00; *p* = 0.970; ns	χ^2^ = 0.00; *p* = 0.961; ns
		adult	χ^2^ = 5.45; *p* = 0.020; *	χ^2^ = 16.92; *p* ≤ 0.001; ***	χ^2^ = 6.53; *p* = 0.011; *
Time spent in the distal quadrant Time spent in the distal quadrant	TXT × STAGE, TXT × ENV × STAGE TXT × STAGE, TXT × ENV × STAGE	juvenile	χ^2^ = 2.91; *p* = 0.088; ns	χ^2^ = 0.58; *p* = 0.444; ns	χ^2^ = 0.43; *p* = 0.512; ns
		adult	χ^2^ = 6.41; *p* = 0.011; *	χ^2^ = 4.09; *p* = 0.043; *	χ^2^ = 11.48; *p* ≤ 0.001; ***
Time spent in the upper zone Time spent in the upper zone	TXT × STAGE TXT × STAGE	juvenile	χ^2^ = 3.10; *p* = 0.078; ns	χ^2^ = 0.02; *p* = 0.878; ns	χ^2^ = 0.77; *p* = 0.380; ns
		adult	χ^2^ = 4.06; *p* = 0.044; *	χ^2^ = 1.77; *p* = 0.183; ns	χ^2^ = 4.09; *p* = 0.043; *
Duration of freezing episodes	ENV × STAGE	juvenile	χ^2^ = 0.28; *p* = 0.599; ns	χ^2^ = 3.94; *p* = 0.047; *	χ^2^ = 3.30; *p* = 0.069; ns
		adult	χ^2^ = 0.00; *p* = 0.990; ns	χ^2^ = 5.32; *p* = 0.021; *	χ^2^ = 0.00; *p* = 1.000; ns

Simple effects by developmental stage for behavioral variables showing significant interactions in the omnibus model. Simple effects by developmental stage are presented only for variables that exhibited a significant interaction with STAGE in the omnibus TXT × ENV × STAGE model. For each variable, a TXT × ENV model was fitted separately within each developmental stage. TXT, pharmacological treatment; ENV, housing environment; CTRL, control group; VPA, valproic acid; EE, enriched environment; SE, standard environment. Inference was based on Type III Wald χ^2^ tests. Significance codes: ns, not significant; * *p* < 0.05; ** *p* < 0.01; *** *p* < 0.001.

## Data Availability

The data presented in this study are available on reasonable request from the corresponding author.

## Abbreviations

The following abbreviations are used in this manuscript:

ASD	Autism Spectrum Disorder
BDNF	Brain-Derived Neurotrophic Factor
dpf	Days Post-Fertilization
EE	Enriched Environment
GLMM	Generalized Linear Mixed Models
HDAC1	Histone Deacetylase 1
hpf	Hours Post-Fertilization
HPI axis	Hypothalamo-Pituitary-Interrenal axis
NLGN3	Neuroligin 3
NRXN1	Neurexin 1
NTT	Novel Tank Test
SE	Standard Environment
SHANK3	SH3 and multiple ankyrin repeat domains
VPA	Valproic Acid
